# Security, privacy, and confidentiality issues on the Internet

**DOI:** 10.2196/jmir.4.2.e12

**Published:** 2002-11-22

**Authors:** Grant Kelly, Bruce McKenzie

**Keywords:** Access to Information, Computer Security, Confidentiality, Data Collection, Information Services, Informed consent, Internet, Organizational Policy, Privacy

## Abstract

We introduce the issues around protecting information about patients and related data sent via the Internet. We begin by reviewing three concepts necessary to any discussion about data security in a healthcare environment: privacy, confidentiality, and consent. We are giving some advice on how to protect local data. Authentication and privacy of e-mail via encryption is offered by Pretty Good Privacy (PGP) and Secure Multipurpose Internet Mail Extensions (S/MIME). The de facto Internet standard for encrypting Web-based information interchanges is Secure Sockets Layer (SSL), more recently known as Transport Layer Security or TLS. There is a public key infrastructure process to `sign' a message whereby the private key of an individual can be used to `hash' the message. This can then be verified against the sender's public key. This ensures the data's authenticity and origin without conferring privacy, and is called a `digital signature'. The best protection against viruses is not opening e-mails from unknown sources or those containing unusual message headers.

## Privacy

`Privacy' is a vaguely defined term that, in an online context, includes the right of an individual to:

- Determine what information is collected about them and how it is used. Sometimes we are not aware what data are being collected about us (e.g. via `cookies' on a Web site--see Glossary) or how it may be used. Registering with a Web site (i.e. giving your name, e-mail address, medical registration number, etc.), for example, may enable that site to keep track of what you--a readily identifiable individual--view or spend online. Such information could be passed on to third parties. Some sites publish `privacy policies' in an attempt to inform users and reduce the chances of patients or healthcare professionals placing their privacy at risk.
- Access information held about them and know that it is accurate and safe.
- Anonymity (e.g. not having your Web-browsing habits tracked).
- Send and receive e-mail messages or other data (e.g. credit card numbers) that will not be intercepted or read by persons other than the intended recipient(s). Encryption (discussed below) is one way of ensuring this.

For more information about privacy on the Internet, see Box 1.

## Statutory and professional considerations

### Confidentiality

The ethical duty of confidentiality is defined by the British Medical Association as `the principle of keeping secure and secret from others, information given by or about an individual in the course of a professional relationship' [1]. In the UK the legal duty of confidentiality is underpinned by the Data Protection Act (1998), regulating the processing of information (`data') that could lead to the identification of individuals--including its collection, storage, and disclosure [2]. To ensure the protection of confidentiality in an electronic environment the General Medical Council (GMC) recommends that doctors should [3]:

- Make appropriate security arrangements for the storage and transmission of personal information.
- Obtain and record professional advice given prior to connecting to a network.
- Ensure that equipment, such as computers, is in a secure area.
- Note that Internet e-mail can be intercepted.

### Consent

`Consent' for our purposes is the means by which we are authorized by an individual to process information about them based on their informed understanding of what we intend.To include identifiable patient information in an e-mail message or on a Web site in the absence of a patient's express consent would constitute a breach of confidentiality. Obtaining consent should involve making the patient aware of any risks to his or her privacy and the arrangements in place to protect it. Identifiable patient information could therefore be transmitted via the Internet with the informed consent of the patient, and with regard for the advice of the GMC (or equivalent professional body) and established principles such as those of Caldicott (see Box 2) and the Data Protection Act (see Box 3).

**Privacy resources on the Internet**

- **Platform for Privacy Preferences Project (W3C):**
http://www.w3.org/P3P/
- **Understanding security and privacy (Netscape):**
- **Privacy and security fundamentals (Microsoft):**
http://www.microsoft.com/privacy/safeinternet/
- **e-Health Code of Ethics (Internet Healthcare Coalition):**
http://www.ihealthcoalition.org/ethics/ehcode.html

**Caldicott Principles**

In relation to identifiable patient information:

- Justify the purpose(s) for using confidential information.
- Only use it when absolutely necessary.
- Use the minimum that is required.
- Access should be on a strict need-to-know basis.
- Everyone must understand their responsibilities.
- Understand and comply with the law.

**For further information, see:**

http://www.doh.gov.uk/nhsexipu/confiden/report/index.htm

**Data Protection Act Principles**

Personal data must be:

- fairly and lawfully processed
- processed for limited purposes
- adequate, relevant, and not excessive
- accurate
- kept for no longer than necessary
- processed in accordance with the data subject's rights
- secure
- not transferred to countries without adequate protection.

For further information, see:

http://www.hmso.gov.uk/acts/acts1998/19980029.htm

Information that cannot result in identification of an individual may have been `anonymized' (where identifiers are removed) or `aggregated' (where data from a number of individuals are summed).The requirement for consent to transmit or place such information online in this event is less certain, but perhaps prudent, although such non-personal data are not subject to legal restriction (i.e. the Data Protection Act).

## Where is the enemy?

Security tends to be the progeny of scandal. A few years ago, a bank in the Midwest USA purchased a hospital along with its medical records. It coolly compared the records against its personal bank accounts, and foreclosed on the loans of all account holders with a diagnosis of cancer. It was business-like, simple, ignorant, cruel, and an example of the damage that medical data can do in the wrong hands. Today computer `security' is typically perceived to mean keeping hackers (those attempting unauthorized computer access) and other troublemakers from your private data. But what if such troublemakers are part of the system, or even own it?

Clearly, a simple `cops and robbers' model does not offer enough protection, highlighting the need to ensure data security at multiple levels. The risks are internal, external, and random, and can result in data damage, falsification, loss, or leakage. It is helpful to imagine your connected system as resembling a data stream right from your keyboard to that of the recipient, and to consider the risks along the way.

## Protecting local data

Even before you connect, your data is at risk. Clearly you don't want your Internet-linked clinical system or home computer to be burnt, flooded, stolen, hit by lightning, damaged by third party software, or accessed by untrained staff or inappropriate people.You will need to back it up properly, look after the backups, and periodically reconstitute the system from backups so that you know it will work if you ever need it.

Ensure that your terminal or PC is left logged out when you are apart from it for a reasonable length of time. Most systems can be set to log out automatically by default under these circumstances and this makes good sense. Make sure that your screen shows information only to people who are entitled to see it.

If you connect to the Internet at work (e.g. via NHSnet) you may wish to ensure that your e-mail server has central control over a shared address book, with limited access rights to alter it and to reply to external addresses. Doing so prevents staff from using e-mail at work to converse with friends--which not only reduces working efficiency, but also provides a means of access for viruses (see below) and other unwelcome material.

Appropriate advice and countermeasures are detailed elsewhere [4-5], enabling you to develop robust protocols to preserve the integrity of your local system. Further NHS-specific guidance is available from the NHS Information Authority Web site: **http://www.standards.nhsia.nhs.uk/sdp/**

## The risks of connecting

### Open systems: the Internet

Linking computers together means that you can access other people's data, but it inevitably follows that this allows others to access data on your own system. Until such time as individual computers or networks are linked together they resemble `islands' of electronic data. Security on a data island is simple: reassuringly firm borders trap all unauthorized entrants. However, when you build bridges by creating a network link this approach on its own is inadequate. When a computer connects to the Internet, it loses its island status by compromising the integrity of its `borders'. Any potential benefits of connecting must be weighed against the risks to your own data. In a healthcare environment, this data is often of a highly sensitive nature. Even connecting a home computer may expose data, such as banking details, which you would prefer to remain private.

### Closed systems: the intranet

Why connect in such an open way? Why not restrict the connection to `friends' only? In other words, why don't we connect only to trusted computers over trusted network links, thus extending our own trusted computing base? Enter the intranet. Intranets are suited to smaller organizations with enforced security policies and strict personnel control--something not always attainable within a large health service.They are by nature restrictive, as security through exclusion conflicts with the potential of a network to enhance medical communications in a connected world. Intranets may provide a false sense of security: as the electronic thief attacks the weakest link in the chain, security measures must reflect this. A properly secured intranet therefore demands such things as locked rooms for terminals, physiological checks for terminal access, and armoured, pressurized cables to detect cable tapping.

### Virtual private networks

Blurring the divide between public and private networks, a virtual private network (VPN) uses a `tunnelling protocol' and encryption (see below) to send private data through public networks such as the Internet. Although communicating parties do not need to invest in a private network infrastructure, they have no control over the network used and no guaranteed standard of service.The lack of interoperable implementations has been the main impediment to the deployment of VPNs to date [6].

### Firewalls

Just as you wouldn't allow anybody to listen in to your telephone conversation, so you need to care for your Web browsing sessions and e-mail exchanges. For this purpose you need a firewall, designed to prevent damage to your system.These software or hardware devices operate by recognizing the IP address that a message or system query comes from, and only allowing past those that are recognized as `good' or trusted. With the advent of higher-risk `always on' Internet connections, firewall solutions of varying complexity are readily obtainable.

## Protecting data in transit

Whether you are connected to NHSnet or the Internet the security threats to your data in transit are the same; data may be subject to loss, late delivery, damage, or attack. Against loss or lateness, there is little the individual can do, but damage or attack can be dealt with.You should assume the wires (or other network infrastructure) could be got at--as indeed they can--and thus must give your data a metaphorical envelope to maintain its integrity and privacy. This is precisely what cryptography can do.

### Message encryption

**Figure 1:**
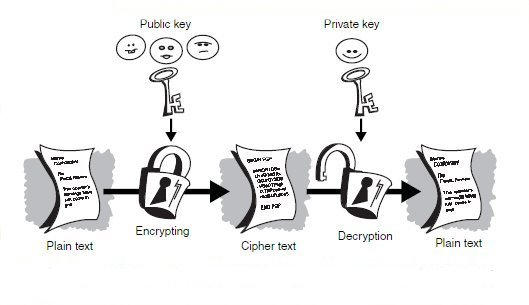
Using a public/private key pair to encrypt messages helps ensure protection during transit

A popular technique for protecting messages in transit is so-called asymmetric public-key infrastructure (PKI) cryptography. Alice and Bob (who wish to exchange messages) each use an algorithm based on very large prime numbers to develop two separate but related numbers, by way of typing in a pass-phrase. Both end up with an alphanumeric code that forms their `public' key (which they publish), and an alphanumeric code that forms their `private' key (known only to themselves and represented by their passphrase). If Alice wishes to send a message to Bob, she finds his public key (typically from a directory), writes her message, and encrypts (addresses) the data to Bob's public key, thus producing a unique set of digital data. Bob receives this in encrypted form and uses his private key to extract the data back into Alice's original text message.This process is illustrated in Figure 1.

In use, this is easier than it sounds, and confers integrity (the data haven't been manipulated), authenticity (the identity of the sender is known), nonrepudiation (the data can't be disowned) and privacy on the data. Any attempt to interfere or damage the contents messes up the mathematics, and the message becomes unintelligible, thus warning the recipient not to trust it. Provided the verification of the identity of the key-holders is carried out in a dictatorial fashion, the origin authentication of the message is also assured. If only Alice knows the private phrase key to make an exchange work, then only Alice can have sent the message.

Authentication and privacy of e-mail via encryption is offered by Pretty Good Privacy (PGP) and Secure Multipurpose Internet Mail Extensions (S/MIME), both proposed Internet standards.

- **Pretty Good Privacy (PGPi Project):**
http://www.pgpi.org/
- **S/MIME (RSA Security Inc.):**
http://www.rsasecurity.com/standards/smime/

### Browser encryption

As we move towards a browser-accessible type of electronic patient record there will arise a need to protect the exchange of data from leakage and attack. A precedent has been set by the widespread practice of Internet banking and commerce, which out of necessity involves transmitting confidential information. The de facto Internet standard for encrypting Web-based information interchanges is Secure Sockets Layer (SSL), more recently known as Transport Layer Security or TLS [7]. SSL/TLS can also be used to encrypt e-mail messages. It uses a symmetrical one-time electronic key that works between the browser and the server for as long as the connection is open. When the session ends, the encryption dies with it, and thus it depends largely on its length of key structure and short time of operation for its safety. SSL/TLS is more demanding on server resources than non-encrypted connections, so secured Web pages are often slow to display.

Assurance of identity (authentication) on the Web presently requires the use of a certificate supplied by a third party Certificate Authority, such as VeriSign Inc.: **http://www.verisign.com/**

UK readers should note that the NHS has its own cryptography strategy: **http://www.doh.gov.uk/nhsexipu/strategy/crypto/index.htm**

## Receiving data

### Digital signatures

**Figure 2:**
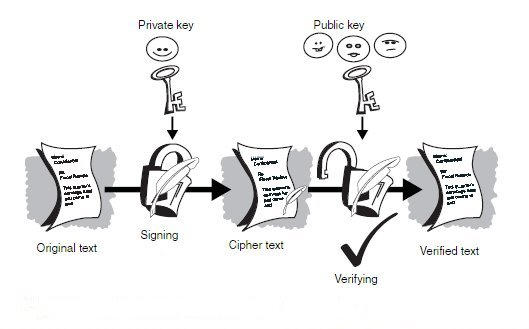
Using a public/private key pair to verify a digital signature

There is a simpler PKI process using the same algorithms referred to above to `sign' a message whereby the private key of an individual can be used to `hash' the message.This can then be verified against the sender's public key. This ensures the data's authenticity and origin without conferring privacy, and is called a `digital signature'.The process is illustrated in Fig. 2. In the UK the Electronic Communications Act 2000 provides the legal framework for the recognition of digital signatures [8].

### What about viruses?

Viruses are small segments of code that have been inserted into computer files, often with malicious intent. An infected file may cause annoyance or the loss of data. In theory, any file you download from the Internet is a potential vector. Viruses may also be present in files attached to e-mail messages (but cannot be transmitted via a text-only e-mail itself ). There are a number of antiviral programs available (some are free) that will screen for and help you neutralize infected files on your computer-- before they are activated or have a chance to `replicate'. Some viruses are activated when you use an infected program; others merely require you to view an infected document.Antiviral programs act like the body's immune system in that they are always on the lookout for `foreign' material--in this case, foreign program code. However, even if your software is regularly updated it won't catch all viruses (especially new ones). Security should be based on the sound sense of not opening e-mails from unknown sources or those containing unusual message headers.

## Conclusions

The protection of personal data in a connected world defaults not so much to high-tech applications or hardware, as to careful management of staff and relatively common techniques to ensure the simple, frequent risks are catered for. The determined criminal or government agency will get access somehow, but what matters to doctors is making sure that we take care of the data we collect about patients in a manner appropriate to the twenty-first century.
